# An Evaluation of the Effectiveness of Local Delivery of Zoledronic Acid in Accelerating Bone Healing After the Extraction of Mandibular Third Molars

**DOI:** 10.7759/cureus.35503

**Published:** 2023-02-26

**Authors:** Bahri Ahmed, Issam Alkhouri, Ahmad Albassal, Asaad Shehada

**Affiliations:** 1 Department of Oral and Maxillofacial Surgery, Faculty of Dental Medicine, Damascus University, Damascus, SYR

**Keywords:** mandibular third molars, bone regeneration, bone density, gelatin sponge, zoledronic acid

## Abstract

Background and objective

Zoledronic acid (ZA) has been reported to aid with the formation of new bone, inhibit osteoclastic bone resorption, and improve osteoblast proliferation. This split-mouth randomized clinical research aimed to evaluate the effect of the local application of ZA on bone regeneration after the removal of bilateral mandibular third molars.

Methods

A randomized, split-mouth study involving 12 patients aged 19-35 years requiring extraction of bilaterally mandibular third molars was conducted. The extraction of mandibular third molars on both sides was conducted in one session for all patients. In each participant, a gelatin sponge (Gelfoam) soaked with ZA was randomly applied to one cavity of the extraction socket. A gelatin sponge soaked with normal saline was applied to the opposite cavity; all patients were blinded as to which socket the drug was applied to. The study was conducted over a period of two months. The changes in bone density (BD) in the socket were assessed through cone-beam CT (CBCT) images; two images were taken for each patient at two different time points: immediately after extraction (T0) and after two months (T1).

Results

BD values in the socket on both sides of extraction increased from T0 to T1. There were statistically significant differences when comparing the amount of change in radiographic BD from T0 to T1 between the two sides of the extraction (p<0.05); the increase in radial BD between the two different time points was more significant in the ZA group.

Conclusions

Within the limitation of this study, the local application of ZA radiographically improved bone healing in a statistically significant manner and could be a cost-effective and simple way to activate bone regeneration.

## Introduction

The regeneration of bone involves a complicated series of events that typically lead to complete structural and functional bone repair [1]. Most bone defects are spontaneously repaired; however, there may be insufficient regeneration during the healing process following pathological events, such as severe trauma, bone tumors, and osteoporosis [2]. Various methods have been developed to enhance the quantity and quality of the repaired bone. In addition to biological mediators, one of the substances used is chemical drugs such as bisphosphonates (Bp), a chemical analog of pyrophosphates. Since its discovery, Bp has been widely used to treat metabolic bone disorders, including osteoporosis, Paget’s disease, multiple myeloma, and hypercalcemia of malignancy. Due to its high binding affinity to hydroxy apatite and its properties by inhibiting bone loss, Bp increases bone mass [3].

However, when used regularly, Bps might cause specific adverse effects. According to the literature, the systematic use of Bp has been associated with initial influenza-like illness, renal failure, and osteonecrosis [3]. However, it has recently been demonstrated that local application, which acts directly on osteoclasts and osteoblasts, is safer and more effective [4]. Zoledronic Acid (ZA) is one of the most potent Bps in clinical practice [5].

Many studies have shown that systemic and local applications of ZA may have positive effects on new-bone formation [4,5]. A single intraoperative dosage of ZA has been shown to have favorable effects on several models of bone healing and repair [5]. It is known as a third-generation Bp that contains a nitrogen atom within its heterocyclic structure [6], which has a crucial role in preventing bone resorption compared to other forms of Bps [7]. It also improves bone density (BD) [8,9]. It is used intravenously at a rate of once a year to treat osteoporosis and reduce the risk of osteoporosis-related fractures in older people [10,11]. These drugs prevent osteoclasts from proliferating and induce cell death of osteoclasts [9,12].

However, in in vitro culture of ZA-loaded membrane, the results have shown that ZA could not only inhibit osteoclastic bone resorption but also improve osteoblast proliferation [13]. In addition, its use on the surface of implants has proven to accelerate the occurrence of osteointegration, as it also accelerates the events of bone healing in cases of distraction osteogenesis [14,15,16].

In this study, we aimed to evaluate the impact of locally administered ZA on bone regeneration.

## Materials and methods

Design and registration of the study

This randomized controlled radiological trial involved 12 patients (nine females and three males) with a mean age of 23.67 years scheduled for extraction of the lower third molar, selected from those visiting the outpatient clinics of the Oral and Maxillofacial Department, Faculty of Dental Medicine, Damascus University from January 2021 to December 2022. This clinical trial was authorized by the Research Ethics Committee of Damascus University (registration no: 2020-3477). The patients' signed agreement to participate in the study was obtained after they had been informed of its objectives and procedures verbally and in writing.

Sample size estimation and eligibility criteria

The total sample size of 12 was calculated by using G.Power 3.1 software and based on the results of a pilot study in six samples where third molar sockets were filled by a sponge with ZA, with 1.91 effect size (mean BD at T0: -330.25 ± 106.67; mean BD at T1: -122.08 ± 63.79) with 95% statistical power and 0.05 α error probability. Based on this, the minimal sample size was deemed to be four.

We clinically and radiographically examined the patients' oral cavity and evaluated the condition of the third lower molars, adjacent teeth, and periodontal tissues.

The inclusion criteria were as follows: (1) male and female patients aged 18-40 years, (2) patients indicated for bilateral extraction of third molars, and (3) patients without systemic diseases that may affect bone healing. The exclusion criteria were as follows: (1) any patients with a history of systemic diseases that could affect bone regeneration, (2) patients who had blood disorders, (3) pregnant patients, (4) smokers, and (5) patients with poor oral hygiene.

Surgical procedures

**Figure 1:**
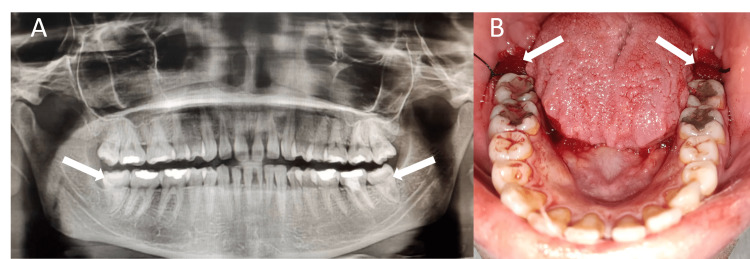
(A) Diagnostic panoramic radiograph. (B) Clinical image after the extraction of bilateral mandibular third molars and the application of ZA-loaded Gelfoam on one side and saline-loaded Gelfoam on the other side

Both lower third molars were extracted on the same day under local anesthesia; the third molars were extracted by a single surgeon (a master’s student in oral and maxillofacial surgery) for orthodontic reasons under all aseptic conditions. Afterward, in each participant, a gelatin sponge (GelSpon® “Bovine Origin”, Eucare Pharmaceuticals, Chennai, India) soaked in ZA (Zoldria, Cipla Limited, Mumbai, India) for five minutes was applied to one cavity of the extraction socket, which constituted the study arm, and a normal-saline-loaded gelatin sponge was applied to the opposite cavity, which constituted the control arm, as randomly selected by a coin-toss method performed by the surgeon’s assistant. Finally, the margins of the gingiva were sutured with 3-0 silk to hold the gelatin sponges (Figure 1).

Postoperative procedures

The patients underwent their first cone-beam CT (CBCT) on the same day of surgery (T0), and we remained in contact with the patients until the follow-up CBCT two months later (T1). According to Peterson’s Principles of Oral and Maxillofacial Surgery - 3rd Edition, radiographic evidence of bone development becomes visible in the sixth to eighth weeks following tooth extraction [17].

**Figure 2:**
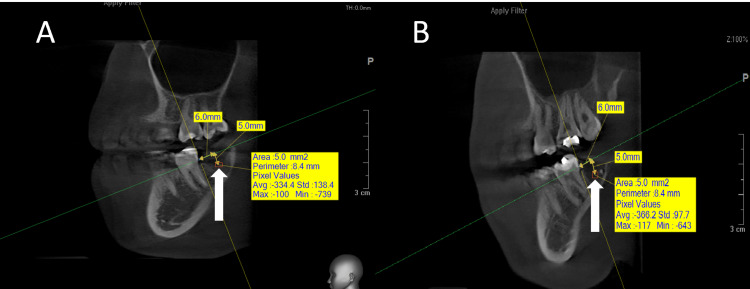
A radiographic image showing the measurement of the bone density in the two study groups at time T0 (day of surgery) (A) The side where ZA-loaded Gelfoam was applied. (B) The side where saline-loaded Gelfoam was used

**Figure 3:**
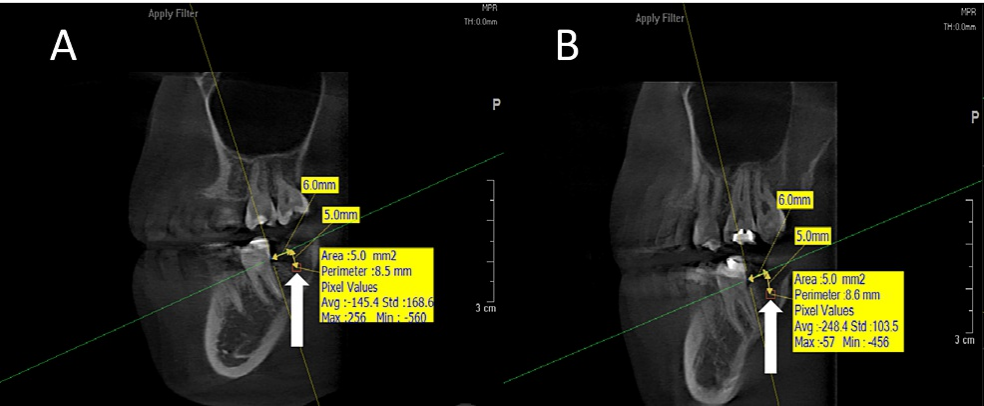
A radiographic image showing the bone density measurement in the two study groups at time T1 (two months after surgery) (A) The side where ZA-loaded Gelfoam was applied. (B) The side where Gelfoam with saline was used

The radiographs were studied to assess the difference in BD between the first-day image and the follow-up, and the results were compared between the two groups (study-control). The BD was analyzed in the radiographs by drawing a square with a specific area in the socket, the center of which was a particular point that was determined by the intersection of three specific levels that could be reproducible in all follow-up images (Figures 2, 3).

Statistical analysis

Statistical analysis was performed with IBM SPSS Statistics v. 20.0 (IBM Corp., Armonk, NY). A p-value of less than 0.05 was deemed statistically significant. Statistically significant differences between intra-group and inter-groups were determined by paired T-test after investigating the normal distribution of the study variables by using the Kolmogorov-Smirnov test with the descriptive statistical analysis.

## Results

Twelve patients (nine females, three males) with 24 alveolar sockets after the extraction of the mandibular third molar were included in this clinical trial, with 12 sockets for every group. Data regarding the age and gender of the patients are presented in Table 1.

**Table 1:** Sample characteristics SD: standard deviation

Frequency and percentage according to gender		
Male		Female
3 (25%)		9 (75%)
Descriptive data according to age, years		
Minimum	Maximum	Mean ± SD
19	35	23.67 ±4.38

Table 2 presents a summary of the intra-group analysis between T0 and T1 for the study and control groups. According to the paired T-test, there were statistically significant differences between T0 and T1 in the study and control groups.

**Table 2:** Comparisons of the bone density values between T0 (day of surgery) and T1 (two months after surgery) in the study and control groups *Statistically significant SD: standard deviation

Group	Time	Minimum	Maximum	Mean ± SD	P-value
Study group	T0	-671.6	-177.4	-357.79 ± 171.75	0.000027*
	T1	-243.4	-7	-94.80 ± 78.63	
Control group	T0	-487.9	-203	-282.18 ± 91.30	0.000174*
	T1	-197	-48	-124.28 ± 50.49	

Table 3 presents a description of the inter-group analysis between the study and control groups, which studied the change in BD between time T0 and T1 and was calculated to investigate the increase or decrease in BD measurement by subtracting T1 from T0. According to the paired T-test, there were statistically significant differences between the study and control groups.

**Table 3:** Comparisons of the bone density values between the study and control groups *Statistically significant SD: standard deviation

Group	Minimum	Maximum	Mean ± SD	P-value
Study group	75.9	486.9	262.99 ± 132.55	0.000085*
Control group	33	369.9	157.90 ± 98.62	

## Discussion

Bone has a natural ability to regenerate itself; there are many clinical uses for inducing bone regeneration, and its importance in maxillofacial surgery has grown tremendously. The need to improve and accelerate bone healing prompted us to expand the use and experience of surgical techniques and drugs. In this study, we aimed to evaluate the effectiveness of using ZA on Gelfoam after the extraction and compared it with the corresponding alveolar cavity that contained Gelfoam only.

The primary objective of any bone-healing procedure is restoring and maintaining mechanical qualities, such as strength, stiffness, and toughness. The two main factors that determine mechanical properties are the density and quality of the bone [18]. Several studies had used radio densitometry to assess bone healing [18]. In our study, the topical application of ZA compared with spontaneous recovery was studied by evaluating the rate of BD between the two sides of the extraction.

Our results showed that there were statistically significant differences at a 95% confidence interval in the rate of the change in BD between time T0 and time T1 on each side of the study for the experimental group (p=0.000027) and for the control group (p=0.000174), meaning that the BD increased in the two sides of the study between time T0 and T1. Also, the results proved that there were statistically significant differences in the grayscale value in the difference between time T0 and time T1, by subtracting T1 from T0, during the bone-healing phase between the two sides of the extraction (experiment and control sides) (p=0.000085).

The results of this study confirmed the positive effect of the topical application of ZA in bone regeneration. Our findings are in line with those of Dundar et al. [4]. In their study, they compared the effect of topical and systemic ZA on bone regeneration when applied in an experimental model of distraction osteogenesis (DO) in rat mandibles, and the results showed that it might be effective in enhancing new-bone formation in the distraction gap in the mandibles. Our results are also consistent with those of Sörensen et al. [1]. In their study, they aimed to find out about the short-term effects of ZA on bone-implant contact (BIC), bone regeneration, and bone area, and their results showed the effectiveness of the ZA in influencing bone regeneration in a way that decreased bone resorption and increased bone regeneration. We also agree with the results of the studies by Küçüktürkmen et al. and Bilston et al. [19,20], where the topical application of ZA helped to improve the quality and speed of bone healing.

Belfrage et al.'s [21] study compared the systemic treatment with various local applications of bisphosphonate to the graft in a bone chamber model. The results showed that while ZA had a high inhibitory impact on bone resorption, there was a limited inhibition on the formation of new bone. This could be attributed to the fact that bone grafts require remodeling processes before the osteoblasts can be activated. When osteoclasts are inhibited, the process of replacing the bone graft with new bone is disrupted.

Within this study, no complications or side effects were seen in any cases of extraction, and a single topical dose of ZA was safe and did not affect the healing process. These results are consistent with the results of the studies by Ying et al. and Israeli et al. [3,22].

The study has some methodological limitations, which are as follows: (1) the local application of ZA was made only in the lower jaw, (2) the study did not include a histological examination, and (3) the study had a short follow-up duration.

## Conclusions

Within the limitation of this clinical trial, our findings revealed that the ZA-loaded gelatin sponge applied in the alveolar socket after the extraction of the mandibular third molar may increase BD (grayscale) value during the bone-healing phase and could be a cost-effective and simple way to activate and hasten bone regeneration. We also found that the local delivery of ZA was safe and did not cause any side effects.
